# Neutrophil Extracellular Traps in Autoimmunity and Allergy: Immune Complexes at Work

**DOI:** 10.3389/fimmu.2019.02824

**Published:** 2019-12-03

**Authors:** Vanessa Granger, Marine Peyneau, Sylvie Chollet-Martin, Luc de Chaisemartin

**Affiliations:** ^1^Département d'Immunologie et d'Hématologie, UF Auto-immunité et Hypersensibilités, HUPNVS, Hôpital Bichat, Paris, France; ^2^Inflammation Chimiokines et Immunopathologie, INSERM UMR996, Faculté de Pharmacie, Université Paris-Sud, Université Paris-Saclay, Châtenay-Malabry, France

**Keywords:** NETs, autoimmunity, anaphylaxis, immune complexes, neutrophils

## Abstract

Neutrophil extracellular traps (NETs) have been initially described as main actors in host defense owing to their ability to immobilize and sometimes kill microorganisms. Subsequent studies have demonstrated their implication in the pathophysiology of various diseases, due to the toxic effects of their main components on surrounding tissues. Several distinct NETosis pathways have been described in response to various triggers. Among these triggers, IgG immune complexes (IC) play an important role since they induce robust NET release upon binding to activating FcγRs on neutrophils. Few *in vitro* studies have documented the mechanisms of IC-induced NET release and evidence about the partners involved is controversial. *In vivo*, animal models and clinical studies have strongly suggested the importance of IgG IC-induced NET release for autoimmunity and anaphylaxis. In this review, we will focus on two autoimmune diseases in which NETs are undoubtedly major players, systemic lupus erythematosus (SLE), and rheumatoid arthritis (RA). We will also discuss anaphylaxis as another example of disease recently associated with IC-induced NET release. Understanding the role of IC-induced NETs in these settings will pave the way for new diagnostic tools and therapeutic strategies.

## Introduction

Neutrophil extracellular traps (NETs) are extracellular chromatin filaments produced upon cell activation and decorated with many proteins normally confined to neutrophil cytoplasm and granules. This process was first described in 2004 as a new mechanism to catch, immobilize, and potentially kill bacteria (1). Subsequently, NETs have been shown to kill several species of bacteria, and rapidly limit the extent of the infection in some models (2–4). However, NET contribution to infectious diseases is double-edged. On the one hand, they may play a major role in defense against pathogens but on the other hand, collateral damage in infected host tissues can be significant, due to proteolytic enzymes release or histone toxicity (5–7). For instance, a deleterious role for NETs have been described in life-threatening infectious conditions such as sepsis or pneumonia-associated acute respiratory distress syndrome (8–12). Interestingly, besides their major pro-inflammatory role, NETs might also be able to downregulate dendritic cell activation and promote Th2 response, thus participating in the resolution of inflammation (13).

In addition to their role during infection, increasing evidence shows that NETosis also happens in a large number of non-infectious inflammation-associated diseases, including various lung diseases, thrombosis, cancer, and auto-immune diseases (14). In the lung, we and others reported that NETs are found in high concentrations in patients with chronic obstruction pulmonary disease or asthma (15–20). In thrombosis, a major interplay between platelets, components of the coagulation system and NETs have been unraveled, leading to the emergence of a new concept named immunothrombosis (21). Finally, as NETs are both pro-inflammatory and composed of many potential autoimmune targets, it has been hypothesized that NETs could be strong inducers of autoimmunity. Indeed, a crucial role for NETs has been described in various autoimmune diseases such as systemic lupus erythematosus (SLE), rheumatoid arthritis (RA), vasculitis or diabetes (22–24).

## Immune Complexes Trigger NET Formation

### NETosis Mechanism

In the initial studies about NETosis, the authors described a relatively long process (several hours) leading to neutrophil lysis and dependent on NADPH oxidase 2 (NOX2) activation (25–27). Fifteen years later, several distinct pathways in response to various triggers have been described making the definition of NETosis even more complex (28). The lytic or suicidal form of NETosis relies on NOX2-derived reactive oxygen species (ROS) release, allowing the liberation of neutrophil elastase (NE) and myeloperoxidase (MPO) from azurophilic granules. Both histone cleavage by NE and their citrullination by peptidyl deaminase 4 (PAD4) have been initially described as required for chromatin decondensation and extrusion out of the cell. However, several intracellular pathways have been described, and some NET release could be NE- or PAD4-independent (29–31). Autophagy is also probably involved, even if there is yet no consensus as conflicting results were obtained (28, 32–34). Very interestingly, quick non-suicidal pathways of NET release have also been described, where neutrophils remain viable, and can still perform functions such as phagocytosis, chemotaxis or dendritic cell activation (14, 35–37). Some studies have also shown NETs composed of mitochondrial DNA (38) but this mechanism requires more investigation. In addition, NOX2–independent pathways have been described, where calcium influx could triggers the activation of mitochondrial ROS (39, 40). This mechanism has been shown in chronic granulomatous disease patients, who have impaired NOX2 activation (41, 42).

The mechanisms of NET formation and release may vary depending upon the initial trigger (23, 43, 44). A large number of triggers have been described, both artificial and physiological. Among them, IgG and IgA immune complexes (IC) have been shown to trigger NETosis in different situations (38, 45–48).

### Immune Complexes Triggering

In this review, we will focus on the role of IC on NET formation. To date, few *in vitro* studies have documented the mechanisms of NET release in response to pre-formed IC in both murine (49, 50) and human neutrophils (49, 51). Moreover, these studies provided conflicting results especially regarding type of FcγR involved or NOX2 requirement. Using soluble bovine serum albumin (BSA)-IgG IC, Chen et al. highlighted some discrepancies between the results obtained in human FcγRIIA transgenic mice and those using blocking antibodies on purified human neutrophils. The first model suggested the importance of human FcγRIIA during *in vivo* NET release whereas the latter supported a role of FcγRIIIB *in vitro*. The differences between the models could explain this discrepancy. Indeed, in transgenic FcγRIIIB^+^/γ^−/−^ mice which do not express FcγRIIA, FcγRIIIB engagement lead to IC clearance. Moreover, blocking FcγRIIA on human neutrophils does not affect receptor intracellular signaling domain, allowing FcγRIIA to transduce signal mediated by FcγRIIIB engagement. Hence, these results support a cooperative role of FcγRIIA and FcγRIIIB, the two activating receptors constitutively expressed on neutrophils, during IC-induced NET-release (49). However, other studies have pointed out an exclusive role of FcγRIIIB in NET release in response to immobilized ICs (51) or by direct receptor aggregation (52, 53). Similarly, the requirement of NOX2-generated ROS in IC-induced NET release is controversial. Although most studies supported a pivotal role of NOX2 (38, 45, 51, 52), two of them provided opposite results despite similar pharmacological inhibitor [diphenyleneiodonium (DPI)] but different concentrations (49, 54). As DPI inhibits a wide variety of NADP-dependent enzymes as well as mitochondrial flavoenzymes (55), DPI-related NET release inhibition only means that ROS (whatever their cellular origin) are important in this process. Additionally, a role of actin cytoskeleton (49), Syk/Src (49–51), and MAPK (49, 51, 52) have been suggested by a few studies whereas the implication of NE/MPO (49, 51) and PI3K/AKT (49, 51, 52) are debated and need confirmation. All these conflicting results regarding the molecular pathways implicated in IC-induced NET release could be explained at least in part by the heterogeneity of the protocols: type of IC (antigenic system, spatial configuration, antigen, and antibody concentrations), pharmacological inhibitors (type and concentration), blocking antibodies [clone, type (Fab or full Ab)], use of human transgenic mice, and method to quantify NETs. Thus, there is a need for recapitulative studies comparing side by side the different triggers and inhibition strategies to obtain a definitive and clear view of IC-induced NET release pathways.

## Immune Complex-Induced NETs Can Trigger and Perpetuate Various Autoimmune Diseases

**Figure 1:**
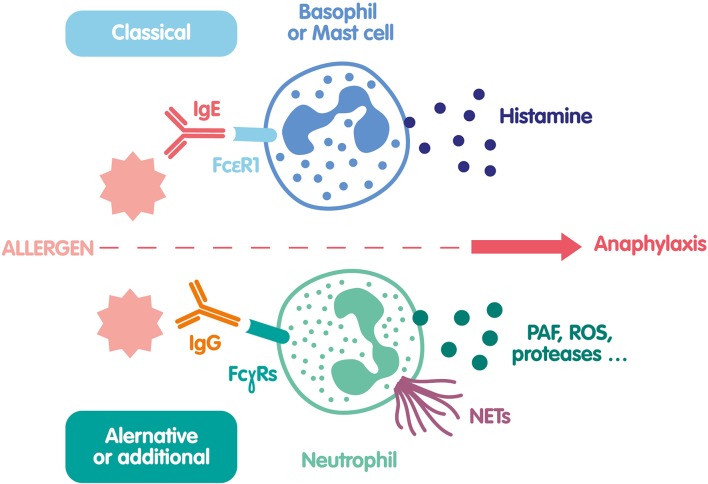
Mechanisms of NET formation in autoimmunity. Autoantigen/IgG IC can bind to several FcγRs expressed at the neutrophil surface and induce their activation. In particular, NOX2 is activated and produce ROS that can in turn activate PAD4 leading to protein citrullination and chromatin decondensation. In parallel, ROS can also help MPO and NE degranulation and translocation to the nucleus contributing to chromatin unfolding. The nuclear membrane breaks down, the decondensed chromatin is released in the cytosol and becomes decorated with various cytosolic and granule-derived proteins. Finally, NETs are released exposing to the immune system a large number of autoantigens that can amplify this mechanism called lytic NETosis. In some conditions, in particular in SLE, these IC can also induce a non-lytic NOX2-independent NETosis via the production of mitochondria-derived ROS and/or DNA; in that case, neutrophils are still alive.

As they expose intracellular endogenous components to the immune system, NETs have been very soon suspected to participate in the initiation of the autoimmune response (Figure 1). Indeed, autoantibodies against several NET components such as DNA, MPO, elastase, citrullinated histones, or proteinase 3 (PR3) are hallmarks of several systemic autoimmune diseases. It has been speculated for several years that anti-ribonucleoprotein (RNP) and anti-DNA antibodies found in the serum of patients with SLE could be produced in response to NET constituents and thus participate to the high level of circulating IC in lupus (56–58). ICs containing self-antigens, in particular RNP/anti-RNP ICs, have been shown to induce NET release, creating an amplification loop where NET components induce autoantibodies leading to ICs, which subsequently trigger NET formation and perpetuate the phenomenon. The role of these IC-induced NETs in the pathophysiology of lupus is not obvious, as the high levels of NETs released during infection does not usually lead to autoimmune response. This suggests that some additional mechanisms must lead to a break of tolerance to NETs. Interestingly, RNP-containing ICs were shown to induce mitochondrial hyperpolarization, increased mitochondrial ROS production and extracellular release of oxidized mitochondrial DNA, a potent proinflammatory compound able to activate type 1 interferon pathway (38). Additionally, self-DNA ICs have been shown to activate plasmacytoid dendritic cells via TLR9, also leading to type 1 interferon release, which has been linked to loss of tolerance (56, 57). An impairment of NETs regulatory mechanisms could also favor loss of tolerance. Some patients with active lupus or lupus nephritis have a deficiency in DNase 1 activity and/or anti-NET antibodies that inhibit DNase effect (23, 59, 60), leading to abnormal NET accumulation. This prolonged presence of NETs could favor rupture of tolerance as well as increase tissue damage (61). Another interesting element is that the composition of NETs from SLE patients is different from that of healthy controls. NETs from SLE patients are richer in toxic compounds (e.g., oxidized alpha-enolase) leading to tissue damage, especially in lupus nephritis (62). Recent observations suggest that several PAD4 polymorphisms are associated to SLE and lupus nephritis, reinforcing the link between NETs and SLE pathophysiology (63, 64). Additionally, in lupus nephritis, circulating IC deposit in the glomerular basement membrane, giving an additional pathogenic role for these IC. Taken together these findings emphasize a major role for IC in the different NETosis pathways involved in SLE, particularly in lupus nephritis (65).

RA is another example where IC-induced NETs are of importance (66, 67). Even if the pathogenesis of RA is not fully understood, many studies have shown that in genetically predisposed patients, anti-citrullinated protein antibodies (ACPAs) play a major role. These autoantibodies target citrullinated self-proteins like histones, vimentin, enolase, collagen, filaggrin, fibrinogen, or calreticulin. Citrullination is a physiological process that occurs in inflammation and during NETosis, due to the activation of PAD4 (66–69). PAD4 activity contributes to RA development, since PAD4-deficient mice have reduced autoantibodies and joint damage in arthritis models (70, 71), and a single nucleotide polymorphism of PAD4 is associated to an increased risk to develop RA in humans (72). Neutrophils from RA patients are activated and produce spontaneously more NETs than healthy donors (73). Additionally, NETosis can be activated by ACPA IgG and IgA ICs (46, 69, 74). Circulating NETs and netting neutrophils in joints are found in patients with RA (69, 75, 76), demonstrating active and widespread NETosis in this context. Thus, IC-induced NETs together with inflammation and synoviocyte activation can enhance the production of citrullinated autoantigens and fuel the autoimmune response, which will in turn produce more ICs (24, 77). Furthermore, synovial fibrocytes can internalize NETs via a RAGE-TLR9 pathway leading to MHC-class II upregulation and presentation to specific T cells of NET-associated citrullinated peptides (78).

The breaking of tolerance to citrullinated proteins is suspected to occur in the airway, in particular in smoker's airway (79–81). Identical citrullinated proteins are present in the joints and in the lung of patients with RA, and high levels of NETs can be found in the sputum of ACPA-positive RA patients, and even in at-risk patients' relatives (positive for HLA-DRB1 allele and ACPA) (82). Nicotine could induce NETs via PAD4 activation (83), and smoking triggered NETosis in several experimental models (84, 85). The link between nicotine, NETs, and loss of tolerance to citrullinated proteins is not fully elucidated yet, but the more recent studies point to an intense lung citrullination process related to high levels of PAD4 and neutrophil activation (22, 86). These NETs can then induce dendritic cell maturation, type 1 IFN release, Th1 expansion, and B cell activation. Furthermore, ectopic lymphoid tissue and high levels of ACPA are observed in the lung of patients with RA reinforcing the idea of a local autoimmune response (82, 87). Finally, the microenvironment, in particular the microbial agents, might themselves play a role in breaking the tolerance; it was for instance recently demonstrated that PAD from *Porphyromonas gingivalis* is able to produce citrullinated proteins and participate to RA pathogenesis (23, 88). Thus, NETs produced in response to infection could constitute in some instances a bridge between infection and autoimmunity. To summarize, NETs are an important source of citrullinated autoantigens in RA, fueling the production of ACPAs in predisposed individuals. They also maintain an inflammatory environment in the lung and in the joints, facilitating neutrophil activation and NET production by the ACPA/citrullinated peptides ICs.

## Immune Complex-Induced NETs Participate to Anaphylaxis

### IgG ICs Formed During Anaphylaxis Induce NET Release

Anaphylaxis is an acute systemic hypersensitivity reaction that can be life-threatening. Because of its extremely fast and unpredictable onset, it is difficult to obtain data on its mechanisms in human, and animal models have been developed to better understand this complex disease (89). The classical pathway is based on the triad IgE/basophil-mastocyte/histamine. During anaphylaxis, cell-surface bound specific IgE on basophils and mast cells react with the allergen and induce the release of preformed mediators such as histamine and proteases, leading to clinical signs of anaphylaxis. However, anaphylaxis can be triggered in mice lacking IgE or their receptor (90, 91), and we reported that up to 30% of patients with neuromuscular blocking agent (NMBA) perioperative anaphylaxis do not have any sign of the IgE pathway (92, 93). An IgE-independent anaphylaxis mechanism has thus been proposed and demonstrated in mice, mediated by neutrophils, specific IgG and FcγRs (94). Specific IgG-IC can bind to various activating FcγRs at the surface of cells such as neutrophils and induce their activation. High circulating levels of several neutrophil-related components and platelet activating factor (PAF) have been described in mice models of anaphylaxis, and in patients experiencing anaphylaxis as markers of neutrophil activation (95–97). The mechanisms of IgG-mediated neutrophil activation during anaphylaxis were first demonstrated in mice models of BSA-induced anaphylaxis. Using depletion and inhibition strategies it was shown that specific IgG-IC binding to neutrophil FcγRIIIA or FcγRIV was sufficient to induce fatal anaphylaxis (94). As human neutrophils do not express these two activating receptors but FcγRIIA, transgenic mice expressing the human FcγRIIA were used to demonstrate a major role for this receptor during anaphylaxis (98, 99). Very recently, these findings were confirmed in an elegant humanized mouse model where the human low-affinity IgG receptor locus, comprising both activating and inhibitory FcγR genes was inserted into the equivalent murine locus (100, 101). The implication of such an IC-mediated anaphylaxis via a new IgG/neutrophil/PAF triad is thus well-demonstrated in animal models and suggested to be relevant in humans by the studies on humanized mice. The existence of this alternative or additional mechanism in humans has been very recently demonstrated in a cohort of 86 patients experiencing NMBA anaphylaxis (93). Blood neutrophils were activated in patients as shown by the upregulation of CD11b, CD18, CD66b, and high levels of circulating elastase. NETosis was also triggered and patients had high levels of circulating NETs remnants (DNA-MPO complexes). Interestingly, a decreased expression of neutrophil FcγRIIA and FCγRIIIB was observed 30 min after anaphylaxis onset. This negative modulation is consistent with the engagement of FcγRs by circulating IC. Moreover, purified anti-rocuronium IgG isolated from a patient could form IC *in vitro* with a rocuronium bioconjugate. These IC were able to activate human neutrophils *in vitro*, and induce NET release (93). Concentration of anti-NMBA IgG and neutrophil activation markers correlated with anaphylaxis severity. This mechanism could be observed in patients lacking any evidence of IgE-dependent anaphylaxis, suggesting that IgG and IgE pathways could be independent, at least in some instances.

### Alternative Mechanisms of NET Release During Anaphylaxis

Besides the role of IgG/IC in NET release during anaphylaxis, one could speculate that other mechanisms exist that could modulate neutrophil activation. Some mediators released both in the acute and the late inflammatory phase of anaphylaxis such as pro-inflammatory cytokines, PAF, or C5a seem able to activate NET release in some conditions, even if there is still conflicting results on the subject (102–105). Recently, a major role of platelets in anaphylaxis has been suggested both in hFcγRIIA transgenic mice model and in humans (106, 107). In the mouse model, the interaction of platelet FcγRIIA with IgG-ICs induced platelet activation/aggregation, whose intensity correlated with the severity of anaphylaxis. Moreover, platelets depletion substantially attenuated symptomatology in mice. As platelets are much more abundant in bloodstream than neutrophils, it seems likely that IgG-ICs interact with platelet FcγRIIA first, before interacting with neutrophils. Activated platelets can aggregate on neutrophils to form platelet-neutrophil complexes detectable *in vivo* during several inflammatory conditions such as sepsis, pulmonary diseases, atherosclerosis (108), and recently allergic shock (106). The formation of these complexes involves the GP1b (glycoprotein 1b)/MAC-1 (macrophage 1 antigen) interaction (108), which is able to induce NET release. Platelets also release several soluble mediators known to activate NETosis (Von Willebrand Factor, platelet factor 4, HMGB1, PAF) (4, 109). Thus, besides direct neutrophil activation by IgG-ICs, other mechanisms involving released mediators or/and activated platelets may contribute to NET release during the acute phase of anaphylaxis.

### Contribution of NETs to Anaphylaxis Mechanism

To date, only one study showed NET formation during anaphylaxis in human (93). Therefore, the pathogenic role of NET in this context is still unclear. However, some hypotheses could be raised according to well-established NET component properties. NET cytotoxicity on vascular endothelium and epithelia has already been shown to be responsible for organ failure in mouse models of sepsis and acute lung injury (4, 5, 110) and may therefore contribute to the pulmonary and vascular symptomatology of anaphylaxis. Whether NETs are formed in the lungs during anaphylaxis has not been directly investigated so far, but interstitial accumulation of neutrophil associated with pulmonary congestion has been demonstrated in a model of casein-induced active anaphylaxis (111). It is also possible that circulating NETs reach lung microcirculation and damage the alveolar-capillary interface as observed in mouse models of acute lung injury (112).

Complement activation is one of the mechanisms implicated in alternative routes of anaphylaxis and in worsening classical IgE-mediated anaphylaxis through C3a and C5a production (89). Along with direct toxicity, NETs could amplify mast cell degranulation by activating the alternative complement pathway (113, 114). Similarly, NETs could amplify bradykinin-mediated circulatory complications through their capacity to activate contact coagulation system (115).

**Figure 2:**
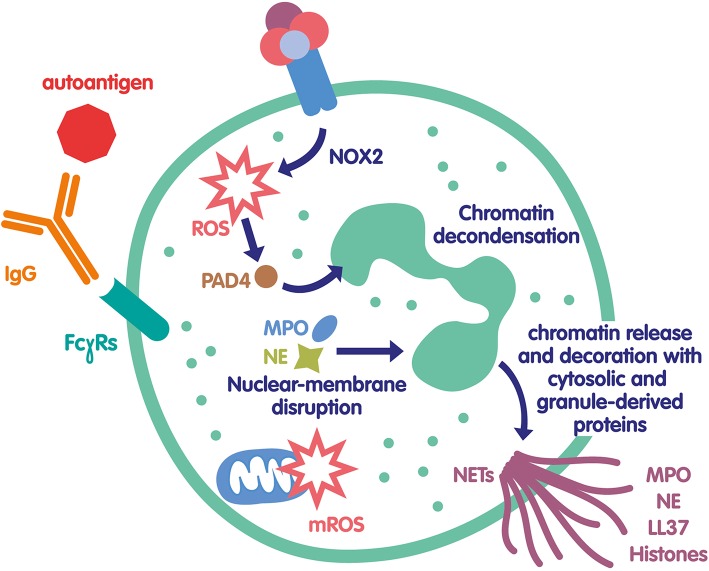
Mechanisms of NET formation during anaphylaxis. The classical pathway of anaphylaxis is based on histamine release by mast cells and basophils activated by the engagement of FcεRI after interaction of specific IgE with an allergen. A second pathway was recently demonstrated both in mice and human. In this pathway the allergen reacts with specific IgG and form an IC that binds to several FcγRs at the neutrophil surface and activate them. In addition to ROS and protease release, neutrophils release PAF and NETs, that could be also involved in anaphylaxis clinical manifestations.

To summarize, very recent human studies have shown that allergen/specific IgG IC are able to activate neutrophils and induce NETs. This new anaphylaxis pathway could participate to clinical manifestations of anaphylaxis (Figure 2), and should be considered in future investigations of diagnostic markers or therapeutic interventions.

## Diagnostic and Therapeutic Perspectives

The implication of NETs in pathology has been prompting several studies investigating its potential as a diagnostic marker or a therapeutic target.

Circulating NETs have been detected in patient's serum in many diseases. Accordingly, NET concentrations have been studied as diagnostic or prognostic markers. Many studies have focused on the concentrations of cell-free DNA or circulating nucleosomes as NET surrogates. However, those are a poor reflect of NETosis since they will be released by any dying cell (116). Some other works focused on citrullinated H3 (H3citr) quantification in serum or tissues. For example, serum H3citr levels predicted the risk of venous thromboembolism in a cohort of 946 cancer patients. Additionally, Jin et al. showed in an interesting study that intratumoral NETs identified by H3citr staining could predict poor survival in post-surgery pancreatic cancer patients (117). The most specific marker of NETs to date are DNA-MPO complexes (116), but fewer studies have investigated their diagnostic relevance. Concentration of DNA-MPO complexes in serum are associated with poor control in asthma (20), severity in anaphylaxis (93), and development of extra-articular nodules in rheumatoid arthritis (118). It could also predict organ dysfunction and 28-day mortality in septic shock (119). High levels of circulating NETs were also associated with poor prognosis in community-acquired pneumonia, though it's not clear exactly which NETs surrogate marker was used in this study (120).

As for therapeutic intervention, two main approaches have been investigated: destruction of NETs or inhibition of their production. NETs can be dismantled by DNAse I treatment or by heparin (121), while their production has been blocked with PAD-4 inhibitors, mostly chloramidine (Cl-Amidine). Disruption of NETs with DNAse I have proven efficient in mouse models of stroke (122), ischemia reperfusion (123, 124), heparin-induced thrombocytopenia (125), and deep vein thrombosis (126). Furthermore, mice treated with DNAse I show less metastasis in mammary tumor models (127, 128). Treatment with chloramidine has shown efficacy in murine models of abdominal aortic aneurysm (122), arterial thrombosis (129), photothrombotic stroke (122), and sepsis (130).

However, studies in pathologies involving IC-induced NETs are scarce. The most relevant works show that pristane-induced lupus is reduced after inhalation of DNAse 1 (131), and arthritis symptoms are reduced by chloramidine treatment in collagen-induced arthritis model (132). Specific inhibition strategies using FcR blocking antibodies represent an interesting possibility, but no study has tested this approach so far, despite the existence of broadly available efficient antibodies.

Globally, while several potential clinical uses of NETs have been described, most results come from mouse models, and large scale clinical trials results are missing.

## Conclusion

ICs can be formed in several clinical conditions. In autoimmunity they can be continuously present in circulation or in tissues, depending on the accessibility of the self-antigen. In contrast, during anaphylaxis they are formed as soon as the allergen enters the body and encounters pre-existing IgGs. As neutrophils express high levels of FcγRs, they can be activated by ICs and release NETs. Beside their tissue toxicity and proinflammatory properties, NETs contain autoantigen and can thus perpetuate autoimmunity. In anaphylaxis, IC-induced NETs release represents a new pathway that may participate in symptoms and severity of the disease. New fundamental and clinical investigations are needed to better elucidate the intracellular mechanisms of IC-induced NET release and evaluate the potential clinical applications of NETs as a biomarker and a therapeutic target.

## Author Contributions

All authors listed have made a substantial, direct and intellectual contribution to the work, and approved it for publication.

### Conflict of Interest

The authors declare that the research was conducted in the absence of any commercial or financial relationships that could be construed as a potential conflict of interest.
